# First Emergence of acrAB and oqxAB Mediated Tigecycline Resistance in Clinical Isolates of *Klebsiella pneumoniae* Pre-Dating the Use of Tigecycline in a Chinese Hospital

**DOI:** 10.1371/journal.pone.0115185

**Published:** 2014-12-12

**Authors:** Xue Zhong, Hongtao Xu, Dongke Chen, Haijian Zhou, Xin Hu, Gang Cheng

**Affiliations:** 1 Department of Pharmaceutics, School of Pharmacy, Shenyang Pharmaceutical University, Shenyang, Liaoning, PR China; 2 Department of Pharmacy, Beijing Hospital, Beijing, PR China; 3 Department of Clinical Laboratory, Beijing Hospital, Beijing, PR China; 4 State Key Laboratory for Infectious Disease Prevention and Control, National Institute for Communicable Disease Control and Prevention, Chinese Centre for Disease Control and Prevention, Beijing, PR China; 5 Collaborative Innovation Centre for Diagnosis and Treatment of Infectious Diseases, Hangzhou, PR China; Iowa State University, United States of America

## Abstract

Tigecycline is one of the few therapeutic options for treating infections caused by some multi-drug resistant pathogens, such as *Klebsiella pneumoniae.* However, tigecycline-resistant *K. pneumoniae* has been discovered recently in China. From 2009 to 2013, nine tigecycline-resistant *K. pneumoniae* isolates were identified in our hospital. Six of nine strains were identified before using tigecycline. To investigate the efflux-mediated resistance mechanisms of *K. pneumoniae*, the expression of efflux pump genes (*acrA*, *acrB*, *tolC*, *oqxA* and *oqxB*) and pump regulators (*acrR, marA, soxS, rarA, rob* and *ramA*) were examined by real-time RT-PCR. Molecular typing of the tigecycline resistant strains was performed. ST11 was the predominant clone of *K. pneumoniae* strains, while ST1414 and ST1415 were novel STs. Efflux pump inhibitor (EPI)-carbonyl cyanide chlorophenylhydrazone (CCCP) was able to reverse the resistance patterns of 5 resistant *K. pneumoniae* strains. In comparison with strain A111, a tigecycline-susceptible strain (negative control), we found that the expression levels of efflux pump genes and pump regulators were higher in a majority of resistant strains. Higher expression levels of regulators *rarA* (2.41-fold, 9.55-fold, 28.44-fold and 18.31-fold, respectively) and pump gene *oqxB* (3.87-fold, 31.96-fold, 50.61-fold and 29.45-fold, respectively) were observed in four tigecycline resistant strains (A363, A361, A368, A373, respectively). Increased expression of *acrB* was associated with *ramA* and *marA* expression. To our knowledge, studies on tigecycline resistance mechanism in *K. pneumoniae* are limited especially in China. In our study, we found that both efflux pump AcrAB-TolC and OqxAB contributed to tigecycline resistance in *K. pneumoniae* isolates.

## Introduction

A derivative of minocycline, tigecycline, is a new class of glycylcyclines. It exhibits a broad-spectrum of activity against most of Gram-positive, Gram-negative pathogens , anaerobes, and ‘atypical’ bacteria [1]. However, tigecycline-resistance has emerged recently in different pathogens such as *Acinetobacter baumannii, Klebsiella pneumoniae* and *Enterobacteriaceae*, especially in multidrug-resistant (MDR) strains. Over the period 2007 to 2013, there have been 13 reports of tigecycline-resistant strains from USA, UK, France, Saudi Arabia, Greece, Spain, Germany and Taiwan and, among them, four were *K. pneumoniae*, seven were *A. baumannii*, one was *Enterobacter hormaechei*, one was *Enterococcus faecalis* and one was *Bacteroides fragilis* [2] . Also, most of them were changed from tigecycline-susceptible to tigecycline-resistant during treatment. The highest minimum inhibitory concentration (MIC) of tigecycline was 24 µg/mL [3]. Recently, emergence of non-susceptibility to tigecycline has also been reported in *A. baumannii* and *K. pneumoniae* in Mainland China [4], [5] . Here, we present nine cases of tigecycline-resistant *K. pneumoniae* in Mainland China. Tigecycline was approved by the US Food and Drug Administration (FDA) in 2005 and was launched in Mainland China in 2011. However, we have discovered six strains resistant to tigecycline isolated before 2011 which shows that there existed tigecycline-resistant strains before using tigecycline. This phenomenon was in accordance with tigecycline-resistant report of Rosenblum et al. which pointed out that tigecycline resistance predates its introduction [6] . We hypothesized that mechanisms of cross-resistance or other as-yet-undefined regulatory elements could be active in such strains. Currently , Veleba M et al. have shown that the factors for decreased sensitivity to tigecycline in *K. pneumoniae* may be contribute to overexpression of the AcrAB RND-type and newly described OqxAB efflux pumps [7]. In addition, the pumps were regulated by *ramA, marA, soxS, rarA* or *rob* of transcriptional activators [6], [8]–[10] . The main purpose of this study was to investigate whether similar mechanisms existed in the resistant strains isolated from Mainland China or whether there are other reasons for this tigecycline resistance.

## Materials and Methods

### Bacterial strains

Two hundred and fifty-four carbapenem-resistant *Enterobacteriaceae* isolates were isolated from clinical samples of Beijing Hospital between 2009 and 2013. (belong to sample secondary use, free of informed consent and ethic). Identification of the strains was performed by using the VITEK-2 and susceptibility testing was carried out by Kirby-bauer, broth microdilution and E-test methods then ten *K. pneumoniae* isolates were selected to study the efflux mechanism. These consisted of nine *K. pneumoniae* isolates resistant to tigcycline and one *K. pneumoniae* isolate, A111, susceptible to tigcycline used as a negative control. *Escherichia coli* ATCC 25922 and *K. pneumoniae* ATCC 11296 were used as reference strains. The strains used in this study are shown in Table 1.

**Table 1:** The characteristics and sources of the clinical isolates.

strain	Isolate date	Source	Type	MLST Type
A103	2008.07.29	Emergency department	Swab	ST378
A352	2009.11.09	Outpatient department	Sputum	ST1414
A361	2009.11.18	Intensive Care Unit	bile	ST11
A363	2009.11.19	Intensive Care Unit	blood	ST11
A368	2009.12.04	Intensive Care Unit	pus	ST11
A373	2009.12.28	Intensive Care Unit	blood	ST11
A840	2012.07.09	Cardiology department	Sputum	ST11
A916	2012.09.04	Emergency department	Urine	ST11
A941	2012.10.10	Respiration department	Sputum	ST1415
A111	2010.4.16	Intensive Care Unit	blood	ST11

Footnote. MLST, Multilocus sequence typing.

### Tigecycline susceptibility testing

Tigecycline susceptibility testing was carried out using the following three different methods: Firstly, the Kirby-bauer method was used as a tigecycline susceptibility test. Then, the minimum inhibitory concentrations (MICs) of tigecycline were determined by standard broth microdilution tests and the E-test (BioMerieux), according to the Clinical and Laboratory Standards Institute (CLSI) recommendation and manufacturers' instructions. The MICs for the strains were interpreted in accordance with FDA guidelines for tigecycline, MIC≦2 µg/mL and ≧8 µg/mL were classified as susceptible and resistant respectively. *E. coli* ATCC 25922 was used as the quality control strain.

### Molecular typing by MLST

In order to check the clonality of the selected isolates,the ten isolates were typed by Multilocus sequence typing (MLST). MLST with seven housekeeping genes (*gapA*, *infB, mdh, pgi, phoE, rpoB* and *tonB*) was performed in accordance with the protocol described on the *K. pneumoniae* MLST database. Alleles and sequence types (STs) of strains were assigned by using tools on the *K. pneumoniae* MLST database. BioNumerics version 5.10 software (Applied Maths, Kortrijk, Belgium) was used to create the minimum spanning tree [11] and in this, the founder ST was defined as the ST with the greatest number of single-locus variants. Types were represented by circles and the size of a circle indicated the number of strains with this particular type.

### Efflux mechanism

The efflux pump inhibitor (EPI) carbonyl cyanide-chlorophenylhydrazone (CCCP; Sigma) was used to investigate the activity of the efflux pump in the tigecycline-resistant and tigecycline-susceptible *K. pneumoniae* isolates. The MICs of tigecycline were determined by the broth microdilution method in the presence and absence of CCCP (fixed concentration of 16 µg/mL). If the MIC values decreased 4-fold or greater in the presence of EPIs, this was defined as a significant inhibition effect [12] .

### Real-time fluorescence quantitative PCR (RT-FQ-PCR)

The expression levels of the *acrR*, *marA*, *soxS*, *rarA*, *rob*, and *ramA* regulator genes and the efflux components *acrA, acrB, tolC, oqxA* and *oqxB* were assessed using RT-FQ-PCR. Oligonucleotide primers and probes used for RT-FQ-PCR (Table 2) were designed with Primer Express Software version 2.0 (Applied Biosystems) and purchased from Life Technologies (AB & Invitrogen). RNA was extracted using an EASYspin RNeasy Kit (Gene-Foci) then, for cDNA synthesis, a cDNA Synthesis Kit (Invitrogen) was used, according to the manufacturer's instructions. The cDNAs were quantified by real-time PCR amplification with specific primers (Table 2) using a Taqman One-Step RT-PCR master mix reagents kit (Life Tech, USA) on a Life Tech 7500 Fast Real-Time PCR System (Applied Biosystems) with an initial incubation at 95°C for 2 min, followed by 40 cycles of 10 s at 95°C, 30 s at 60°C, and 10 s at 72°C. Each sample was processed in triplicate. In all cases, a housekeeping gene (*rpoB*) was used to normalize the expression of the target genes for each isolate. The critical threshold cycle (C_T_) numbers were determined by the detection system software. The amount of target is given as 2^−△CT^. Expression analysis was carried out to measure the relative expression of the mRNA compared with that of *K. pneumoniae* A111.

**Table 2:** Primers and fluorescent probes used for PCR.

Gene	Forward primer	Reverse primer	Fluorescent probe
*ramA*	TGCATCAACCGCTGCGTAT	GCGAATCAAAGCCATACTTGAG	TCGAAATGGCATCTGCAACGGCT
*marA*	GGAGATTGCGCAGAAGCTCA	GGATTCGCCAGGTACATTCG	CCAATCCTGTACCTGGCGGAACGC
*soxS*	GTGGTACCTGCAGCGGATGT	CGGAGATCTGATGGCGATAGTC	CTATATTCGCCAGCGCCGCCTG
*rarA*	CGATTGCCTCGGAAGATGTC	GCGCAGGCCAGATAGACAAT	CGTGGGACTGCTTTACCCGCGTC
*robA*	TATTCTATACCACCGCGCTGAC	GTGCCGTAGACGGTCAGGAT	CCTGCAGCATGCCCATCCAGTG
*acrA*	GCCTATCGCATTGCGGAAG	TTGGCGCTGTCATAGCTGG	TAGCGATATCCAGGCCGGCGTCT
*acrB*	GGAAGCGATCATCCTGGTGTT	ACCGCGAAGGTGCCTAACA	TTCCTGCAGAACTTCCGCGCCA
*tolC*	TACCAGCAGGCACGCATCA	GCTGTCGCGATAGCCATTGT	CGCGCAGTCCATTACTGCCTCAGC
*oqxB*	ATCAGGCGCAGGTTCAGGT	CGCCAGCTCATCCTTCACTT	CAGGCGGAAGCGCGTCTGC
*oqxA*	CGCAGCTTAACCTCGACTTCA	ACACCGTCTTCTGCGAGACC	CCTATTGACGGCCGCGCCAG
*acrR*	CCTGGCGAGTTATGAGCGTAT	GGTAGCTGCGCATTAACACG	TGCATCGCGGCGAAGCTGC
*rpoB*	GGTAATTCCGAGCTGCAATACG	CGCGCTCGTAGATCACCAG	CGTCTGGGCGAACCGGTTTTTGAC

Footnotes. TaqMan probe labeled with an FAM dye at the 5′ end and a minor groove binder (MGB) and nonfluorescent quencher (NFQ) at the 3′ end.

## Results

### Tigecycline susceptibility

254 carbapenem-resistant *Enterobacteriaceae* isolates included *K. pneumoniae* (192 isolates) , *Escherichia coli* (41 isolates) , *Enterobacter cloaca* (12 isolates) and *Enterobacter aerogenes* (9 isolates). Nine tigecycline-resistant *K. pneumoniae* isolates were obtained, a tigecycline nonsusceptibility rate of 4.69% (9/192) for carbapenem-resistant *K. pneumoniae*, One *Enterobacter cloacae* and one *Enterobacter aerogenes* besides *K. pneumoniae* also showed resistance to tigecycline but not the aim of this study. Of all the clinical carbapenem-resistant *K. pneumoniae* isolates, the tigecycline MIC ranged from 0.5–16 µg/ml with an MIC_50_ of 4 µg/ml and an MIC_90_ of 16 µg/ml. Nine *K. pneumoniae* clinical isolates were collected that required a tigecycline MIC over 8 µg/ml (table 3) . The highest MIC value of tigecycline was 16 µg/ml (A361, A363, A368, A373) . In addition, an ordinary clinical strain A111 which was an ST11 type and susceptible to tigecycline was designated a negative control. So, nine resistant strains and one susceptible strain were included in this study. The susceptibility profiles of the ten strains are presented in Table 3.

**Table 3:** Susceptibility profiles of nine tigecycline-resistant and one tigecycline-susceptible strain.

Numbers of stains	KB(cm)	BMD(µg/mL)	E-test(µg/mL)
ATCC 25922	25	0.125	0.125
A103	6	8	16
A352	12	8	8
A840	14	8	6
A916	13	8	6
A941	14	8	8
A363	9	16	16
A361	10	16	16
A368	9	16	16
A373	10	16	16
A111	19	2	2

Footnote. KB, Kirby-bauer; BMD, broth microdilution; MIC, minimum inhibitory concentration(test by BMD and E-test method) ; ATCC 25922 quality control strain KB value range from 20–27 cm, MIC range from 0.03–0.25 µg/mL.

### Molecular typing of the strains

MLST analysis of nine isolates showed four different sequence types (STs). ST11 was the predominant ST, accounting for 6 (70%) of the isolates. Other three isolates belonged to ST378, ST 1414 and ST1415, with 5, 4 and 5 loci different from ST11, respectively. ST1414 and ST1415 were novel STs identified for the first time in this study.

### Efflux pump activity

The effect of the EPIs on tigecycline MICs is shown in Table 4. One strain (A103) showed an 8-fold decrease, four strains (A363R, A361, A368, A373) showed a 4-fold decrease, four strains (A352, A840, A916, A941) showed a 2-fold decrease in the tigecycline MIC in the presence of CCCP (16 µg/ml). There was no reduction in MIC observed in strain A111 under the same conditions.

**Table 4:** The MIC of tigecycline (TGC) in the multidrug resistant strains, with or without an efflux pump inhibitor, Carbonyl hydrogenated chlorophenyl hydrazone (CCCP).

Numbers of stains	MIC/(µg•ml^−1^)	
	TGC	TGC+CCCP
ATCC 25922	0.125	0.125
A103	8	1
A352	8	4
A840	8	4
A916	8	4
A941	8	4
A363	16	4
A361	16	4
A368	16	4
A373	16	4
A111	2	2

Footnote. MIC, minimum inhibitory concentration; TGC, tigecycline; CCCP, carbonyl cyanide chlorophenylhydrazone. ATCC 25922 was used as negative quality control strain.

### Analysis of target pump genes and regulator expression

**Figure 1:**
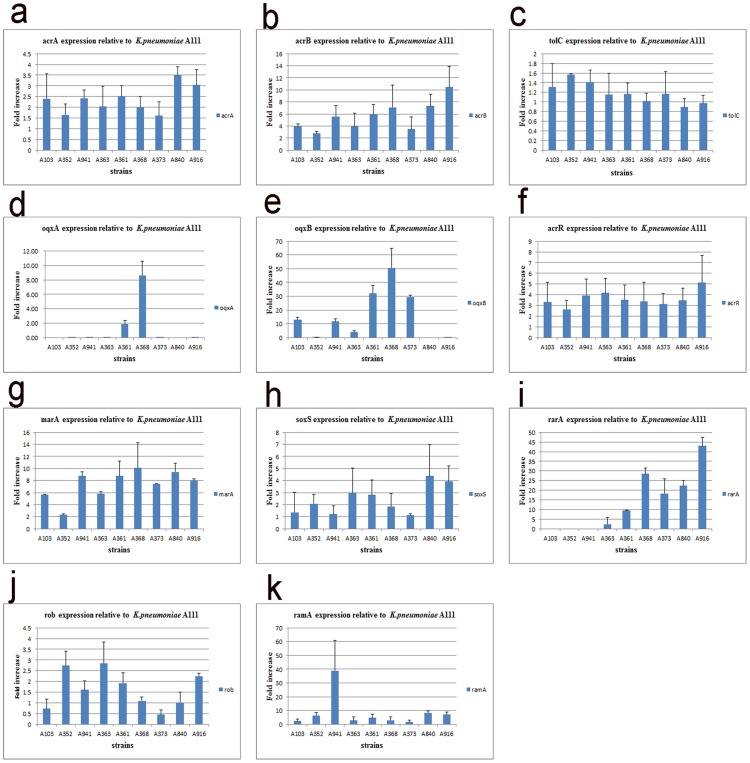
(a-k) Bar chart showing levels of pump genes and pump regulators in clinical strains of *K. pneumoniae*. Relative x-fold increases in the pump genes and regulator levels were quantified after comparisons with *K. pneumoniae* A111 (tigecycline-susceptible).

In our study, nine tigecycline-resistant strains were demonstrated to carry efflux pump genes and pump regulators. The relative x-fold increases in the pump genes and pump regulator levels were quantified after comparisons with *K. pneumoniae* A111 (Fig. 1 a-k). Compared with isolate A111, a higher expression level of regulator *rarA* (2.41-fold, 9.55-fold, 28.44-fold, and 18.31-fold, respectively) and pump gene *oqxB* (3.87-fold, 31.96-fold, 50.61-fold, 29.45-fold, respectively) was observed in four tigecycline-resistant strains (A363, A361, A368, A373, respectively). These data suggested that there was a correlation between the increased expression of *oqxB* and *rarA*. In addition, except for *oqxB*, the amount of *acrB* transcript of the resistant strains was higher in comparison with strain A111. The expression of *acrB* had an increased tendency with *ramA* and *marA* expression. Interestingly, the transcriptional levels of the *soxS* gene, *acrR* gene, and *rob* gene, appeared to be linked to *acrB* expression, but the role of the three genes was not significant (data shown in Fig. 1).

## Discussion

Tigecycline has been developed and has attracted much attention as the agent of last resort to treat clinical infections caused by multidrug-resistant *K. pneumoniae*. In 2011, tigecycline was first launched in Mainland China. However, we discovered six strains which were resistant to tigecycline before 2011, but it cannot be attributed to the use of tigecycline. We hypothesized that this resistance could be indirectly attributed to the use of other antibiotics which were also transported by the same efflux pump because tigecycline is a substrate of chromosomally encoded resistance-nodulation-division efflux pumps. A possible assumption was that these efflux systems were widely existing among *K. pneumoniae*. When strains exposed to some antibiotics which were substrates of efflux pumps, this might lead to overexpression of these efflux pumps. In addition to our report, other recent reports have described cross-resistance to tigecycline. Hornsey et al. and Deng et al. reported the emergence of resistance to tigecycline associated with other antibiotics rather than tigecycline [4], [13]. In addition, another study concluded that the use of carbapenems might induce resistance not only to carbapenems but also to many other antibiotics, including tigecycline [14] . These reports confirmed our assumption that our isolated tigecycline-resistant strains were likely related to overexpression of the efflux pump which caused cross-resistance.

It has been reported that antibiotic-resistance appeared to be mediated in part by active efflux systems [15], [16]. Recently some studies have demonstrated that efflux pump inhibitors (EPIs) are able to reverse resistance patterns by blocking bacterial pumps and preventing discharge of certain antibiotics [16]. In order to detect if there is an overexpression efflux in resistant *K. pneumoniae* strains, we used efflux pump inhibitors (EPIs) CCCP to assess the activity of the efflux pumps. In our study, CCCP restored the susceptibility in five strains (A103, A361, A363, A368, A373), indirectly proving that efflux pump overexpression contributed to tigecycline resistance. However, no significant reduction in tigecycline MICs was observed in the other four strains (A352, A840, A916, A941), indicating that CCCP was not effective. One possible explanation is that EPIs have different specificities towards various efflux pumps and interfere with them in a different manner. It may be that other EPIs, such as PAβN, NMP, reserpine or verapamil, could restore the susceptibility of these strains. Also, the results (isolates A352, A840, A916, A941) of efflux pump activity (using CCCP) as well as the expression levels of target pump genes and its regulators suggested that it might have other mechanisms other than the overexpression of efflux pumps mediating tigecycline resistance.

It has been reported that resistance to tigecycline appears to be mediated in part by the active efflux systems AcrAB-TolC [8], [17]. Also, the AcrAB-TolC pump is activated by several transcriptional regulators such as *ramA, marA, soxS, rob* or *acrR*. These pump regulators play a role in promoting tigecycline resistance due to up-regulation of the efflux pump AcrAB-TolC [9], [10], [18]. Our present study showed that the increased MIC for *K. pneumoniae* strains correlated with the overexpression of *ramA* or *marA*, demonstrating that *ramA* was not always needed to confer tigecycline-resistance, *and marA* was also a global activator of the *acrB* transporter (Fig. 1). A similar hypothesis was proposed recently, some studies have suggested that other pathways to tigecycline resistance must exist in *K. pneumoniae* [6], [7]. One of these studies demonstrated that a newly described OqxAB efflux pump could contribute to tigecycline resistance in *K. pneumoniae* although the chromosomally encoded *rarA* regulator lies downstream of the efflux pump OqxAB [7] . In our study, the strains A363, A361, A373, and A368 had MIC values of 16 µg/ml, and quantitative real-time PCR analyses showed that *rarA* overexpression was observed in conjunction with an elevated expression of the multidrug efflux pump OqxAB, confirming that *raA* might be involved in the regulation of OqxAB production. The results presented here further support a previous report that *rarA* is one of the regulator pathways that controls the expression of *oqxB* in *K. pneumoniae* [19]. However, for the strains A840 and A916, although the expression level of *rarA* was higher, this did not lead to overexpression of *oqxB*; on the contrary, for the strains A941 and A103, the amount of the *oqxB* transcript was higher, but the expression level of *rarA* was not increased in comparison with the susceptible strain A111. This data indicates that some regulators other than rarA may be able to contribute to *oqxB* expression. In addition to our report, one recent report has described the same results. In that study, no differences in the sequences 5′of *oqxA* were found between two strains, which differed 20-fold in levels of *oqxB* transcripts. Thus, they hypothesized the increased expression of *oqxB* in the two strains appears not to be due to mutation in a putative promoter, but might relate to differences in other as-yet-undefined regulatory elements in these strains.[20] We hypothesized that this was the reason why the MICs of four strains (A840, A916, A941, A103) were lower than those of the other strains (A363, A361, A368, A373). Efflux pumps AcrAB-TolC play an important role in strains with MICs of 8 µg/ml (A840, A916, A941, A103) . For the strains with an MIC of 16 µg/ml (A363, A361, A368, A373) , the AcrAB-TolC and OqxAB efflux pumps both contributed to the tigecycline-resistance. From our findings, it was clear that *rarA* as well as those of the pump gene *oqxB* were more important in the case of our selected tigecycline resistant *K. pneumoniae* strains. This result was especially important with regard to the previous observations showing that a high-level of tigecycline resistance was exhibited due to alternative pathways in *K. pneumoniae* [7] . Irrespective of the nature of the pump acting in these strains, we have clearly demonstrated that the efflux pump plays a key role in the tigecycline-resistance in *K. pneumoniae*. However, the mechanisms responsible for the high expression level of *oqxB* leading to tigecycline-resistance remain unknown and need further study.

MLST was performed for molecular typing in the present study. ST11 is the epidemic clone of *K. pneumoniae* carbapenemase 2-producing *K. pneumoniae* in China, and it had only one of the single-locus variants (SLVs) of ST258 which is the dominant clone in Eastern countries [21]. Other three STs had five to six loci different from ST11.

## Conclusion

In conclusion, our study report mechanism of tigecycline-resistant *K. pneumoniae* in Mainland China. The results of molecular typing suggest that the predominant clone of *K. pneumoniae* strains was ST11. In addition, we identified two novel STs, namely ST1414 and ST1415. Efflux-mediated mechanisms, including high expression of AcrAB-TolC and OqxAB efflux pumps appears to play a key role in tigecycline resistance while the pump regulators, *acrR*, *marA*, *soxS, rarA, rob* and *ramA*, all make their individual contributions to the overexpression of AcrAB-TolC and OqxAB efflux pumps. In addition, *rarA* as well as the pump gene *oqxB* are more important in our selected high level tigecycline-resistant *K. pneumoniae* strains. Efflux pump inhibitors (EPIs)-CCCP was able to reverse resistance patterns in the majority of *K. pneumoniae* strains. Although tigecycline is a promising antibiotic for the treatment of infections caused by multidrug-resistant *K. pneumoniae*, the development of tigecycline resistance is an issue of great concern.
